# Charting the equine miRNA landscape: An integrated pipeline and browser for annotating, quantifying, and visualizing expression

**DOI:** 10.1371/journal.pgen.1011835

**Published:** 2025-09-05

**Authors:** Jonah N. Cullen, Jakub Cieslak, Jessica L. Petersen, Rebecca R. Bellone, Carrie J. Finno, Ted S. Kalbfleisch, Kirstine Calloe, Stefano Capomaccio, Katia Cappelli, Stephen J. Coleman, Ottmar Distl, Sian A. Durward-Akhurst, Elena Giulotto, Natasha A. Hamilton, Emmeline W. Hill, Lisa M. Katz, Dan A. Klaerke, Gabriella Lindgren, David E. MacHugh, Mariusz Mackowski, James N. MacLeod, Julia Metzger, Barbara A. Murphy, Ludovic Orlando, Terje Raudsepp, Maurizio Silvestrelli, Eric Strand, Teruaki Tozaki, Dagmar S. Trachsel, Laura S. Valderrama Figueroa, Brandon D. Velie, Claire M. Wade, Bianca Waud, James R. Mickelson, Molly E. McCue

**Affiliations:** 1 Department of Veterinary Population Medicine, College of Veterinary Medicine, University of Minnesota, St. Paul, Minnesota, United States of America; 2 Department of Genetics and Animal Breeding, Poznan University of Life Sciences, Poznan, Poland; 3 Department of Animal Science, University of Nebraska-Lincoln, Lincoln, Nebraska, United States of America; 4 Department of Population Health and Reproduction, School of Veterinary Medicine, University of California - Davis, Davis, California, United States of America; 5 Veterinary Genetics Laboratory, School of Veterinary Medicine, University of California - Davis, Davis, California, United States of America; 6 Department of Veterinary Science, Martin-Gatton College of Agriculture, Food, and Environment, University of Kentucky, Lexington, Kentucky, United States of America; 7 Department of Veterinary and Animal Sciences, University of Copenhagen, Frederiksberg, Denmark; 8 Sport Horse Research Centre, Department of Veterinary Medicine, University of Perugia, Perugia, Italy; 9 Department of Animal Sciences, Colorado State University, Fort Collins, Colorado, United States of America; 10 Institute of Animal Genomics, University of Veterinary Medicine Hannover, Hannover, Germany; 11 Department of Veterinary Clinical Sciences, College of Veterinary Medicine, University of Minnesota, St. Paul, Minnesota, United States of America; 12 Department of Biology and Biotechnology, University of Pavia, Pavia, Italy; 13 Equine Genetics Research Centre, Racing Australia, Scone, New South Wales, Australia; 14 UCD School of Agriculture and Food Science, University College Dublin, Belfield, Dublin, Ireland; 15 UCD School of Veterinary Medicine, University College Dublin, Belfield, Dublin, Ireland; 16 Department of Animal Biosciences, Swedish University of Agricultural Sciences, Uppsala, Sweden; 17 Department of Biosystems, Center for Animal Breeding and Genetics, KU Leuven, Leuven, Belgium; 18 UCD Conway Institute of Biomolecular and Biomedical Research, University College Dublin, Belfield, Dublin, Ireland; 19 Department of Veterinary Science, Gluck Equine Research Center, University of Kentucky, Lexington, Kentucky, United States of America; 20 Centre for Anthropobiology and Genomics of Toulouse, CNRS, Université de Toulouse, Toulouse, France; 21 College of Veterinary Medicine and Biomedical Sciences, Texas A&M University, College Station, Texas, United States of America; 22 Faculty of Veterinary Medicine, Norwegian University of Life Sciences, Aas, Norway; 23 Genetic Analysis Department, Laboratory of Racing Chemistry, Utsunomiya, Tochigi, Japan; 24 Equine Genetics & Genomics Group, School of Life and Environmental Sciences, University of Sydney, Sydney, New South Wales, Australia; 25 School of Life and Environmental Sciences, University of Sydney, Sydney, New South Wales, Australia; 26 Sydney School of Veterinary Science, University of Sydney, Sydney, New South Wales, Australia; 27 Department of Veterinary and Biomedical Sciences, College of Veterinary Medicine, University of Minnesota, St Paul, Minnesota, United States of America; Royal Veterinary College, UNITED KINGDOM OF GREAT BRITAIN AND NORTHERN IRELAND

## Abstract

MicroRNAs (miRNAs) are essential regulators of gene expression, yet few comprehensive databases exist for miRNA expression in non-model species, limiting our ability to characterize their roles in gene regulation, development, and disease. Similarly, isomiRs - length and sequence isoforms of canonical miRNAs with potentially altered regulatory targets and functions - have received even less attention in non-model species, including the horse, leaving a critical gap in our understanding of their biological significance. To address these challenges, we developed an open-source, containerized pipeline for identifying and quantifying miRNAs and isomiRs (FARmiR: Framework for Analysis and Refinement of miRNAs), and an associated interactive browser (AIMEE: Animal IsomiR and MiRNA Expression Explorer). AIMEE was developed to make miRNA expression data more accessible and user-friendly, a feature often lacking from other expression atlases. These tools were developed using equine data but can be readily extended to other species. Using these tools, we aggregated 461 small RNA-seq datasets, spanning 61 distinct tissues, integrating data from public repositories, an American Quarter Horse cohort, and the Functional Annotation of ANimal Genome (FAANG) consortium Thoroughbred samples, predicting 5,781 miRNAs and isomiRs. This work represents the largest systematically curated atlas of equine miRNA expression to date, providing a valuable resource that will enhance our understanding of miRNA and isomiR functions in tissue-specific regulation and ultimately improve biomarker discovery, functional genomics, and precision veterinary medicine.

## Author summary

MicroRNAs (miRNAs) play a critical role in gene regulation, influencing many biological processes including development, metabolism, and disease. Despite their importance, miRNA research in non-model species like the horse has remained limited. Moreover, what research has been done has been more narrowly focused on canonical miRNA expression in specific tissues under various disease states. To address this gap, we developed a pipeline and interactive browser for reproducibly quantifying and visualizing miRNA expression. Using these tools, we aggregated and analyzed 461 small RNA sequencing datasets from 61 equine tissue types, generating the most comprehensive equine miRNA expression atlas to date. Our findings highlight the complexity of miRNA and isomiR expression across tissues and delivers a user-friendly resource for researchers studying gene regulation. Our work enhances the ability to investigate miRNA-regulated biological processes in the horse and provides a framework for similar studies in other species.

## Introduction

MicroRNAs (miRNAs) are a class of endogenous small (~22 nucleotides) noncoding RNAs that primarily regulate gene expression through binding messenger RNA (mRNA), triggering mRNA degradation or the inhibition of translation [1]. Over 60% of human protein-coding genes contain at least one miRNA binding site, highlighting the importance of miRNA-mRNA pairing and miRNA-based regulation [2]. MicroRNAs play critical roles in numerous biological processes, including development [3], stem-cell maintenance [4], metabolism [5], apoptosis [6], and cell-cell communication [7]. Given their large regulatory footprint, miRNA dysregulation has been implicated in many diseases [8–12], and miRNA profiling has demonstrated both ubiquitous and tissue-specific expression across species [13–16].

High-throughput sequencing (HTS) for profiling of human small RNA expression has identified miRNA isoforms (isomiRs) that differ from the reference miRNA (Ref-miR) [17]. Originally believed to be sequencing or alignment errors, isomiRs are now thought to be the result of imprecise cleavage or post-transcriptional modification [18–21], resulting in differences in sequence composition and/or length from the reference form [22–24]. IsomiRs can be categorized into five classes based on compositional differences from their Ref-miR counterparts: reference or canonical (identical to the miRNA database sequence [25,26]), 5’ isomiR (length changes to the 5’ end), 3’ isomiR (length changes to the 3’ end), polymorphic (sequence modifications with identical length), and mixed (length and sequence changes) [27,28]. The region of the miRNA critical for mRNA target recognition is referred to as the “seed sequence”. IsomiRs with edited or shifted seed regions (e.g., 5’-isomiRs or polymorphisms within residues 2–8) are of particular interest [29,30], as they may have altered target sets that impact similar or different biological pathways as compared to their Ref-miR counterpart [31–36]. Similar to Ref-miRs, isomiRs can be highly expressed [28] with tissue-dependent expression patterns [37,38] and have been associated with various disease states, highlighting their potential as biomarkers [39–41]. Despite the availability of many isomiR-capable processing tools [42], “gold standards” do not exist for isomiR profiling. As a result, isomiRs are often excluded from miRNA studies and databases, introducing profiling biases and missing biological significance [43]. While several miRNA databases exist, only a few include isomiR expression [38,44], and, with a few exceptions, these are limited to humans and mice [45].

Characterization of the tissue-specific miRNA transcriptome in companion animals and livestock has lagged behind that of humans and model organisms for several reasons. First, miRNA profiling is often based on the available Ref-miR sequences from databases such as miRBase (v22) [25], and the more sparse annotation of miRNAs in these species may have a significant impact on expression profiling. For example, the current version of miRBase contains 2,656 mature miRNA sequences in humans and 1,978 in mice, compared to only 690 in horses. Of the miRBase horse miRNAs, over half (359) are based on a single *in silico* study [46], and the remainder (331) are from small RNA-sequencing (RNA-seq) of testes from one horse [47]. Moreover, while earlier versions of MirGeneDB (2.1) [26] did not include horse, the latest release (3.0) [48] provides a database of manually curated, high-confidence miRNAs for EquCab3. While this represents a major improvement in annotation quality over previous resources, it is more conservative in scope, containing 438 equine mature miRNA sequences from 417 precursors (69 of which are not in miRBase). As the goal of this study was to catalog all potentially expressed miRNAs, we included the broader set of miRBase sequences. Second, few large-scale miRNA expression profiles of normal equine tissues exist. Most of the equine miRNA efforts have focused on profiling select tissues from horses with or without disease [49–53], during exercise [54–56], or at different developmental stages [50]. MiRNA expression profiling across healthy tissues has been previously conducted with only limited tissue sets [57]. The largest study targeted nine tissues, including gluteus medius muscle tissues (the largest muscle in horses, involved in hip extension) from normal horses and horses with polysaccharide storage myopathy type I (PSSM1) [58]. While these studies identified tissue-specific reference and putative novel miRNAs, isomiRs were not thoroughly characterized. To the best of our knowledge, only two equine miRNA analyses have included isomiRs [59,60]. Third, miRNA data can be difficult to access for animal researchers intending to explore and utilize previous findings, and analyses of miRNA data requires bioinformatics proficiency. Finally, the majority of available computational tools and pipelines for characterizing isomiRs were developed for processing human data, many of which require additional informatic manipulations to configure for non-model organisms [42].

To address these limitations, we developed the Framework for Analysis and Refinement of miRNAs (FARmiR) pipeline and Animal IsomiR and MiRNA Expression Explorer (AIMEE) browser. We used these tools to catalog the most comprehensive collection of miRNA expression at the isoform resolution in normal equine tissues, resulting in the identification of 5,463 isomiRs. These resources allow for the reproducible processing and analysis of high-throughput small RNA-seq datasets, and the export of raw and normalized data for additional downstream analyses. The aggregated expression data, together with the pipeline and browser, will be a significant resource for researchers investigating the composition and functional roles of miRNAs in species that lack expression databases like those available for humans and mice. Finally, our pipeline-browser framework will enable researchers to process their own RNA-seq samples in a consistent and reproducible manner, facilitating comparisons across different disease states, phenotypes of interest, and developmental processes with our comprehensive catalog.

## Results

### Equine miRNA expression atlas

#### Overview and QC of the primary, FAANG, and public small RNA-seq datasets.

A total of 5.9 billion (B) raw reads were obtained from 230 *primary* samples sequenced as part of this study, 86 *FAANG* samples [61,62], and 185 *public* samples (S1 Fig). The 185 public samples were sourced from 14 distinct BioProjects. Of these, 11 projects included a single tissue type, 1 project included two tissues (uterus and uterine fluid), and two projects contained samples from four tissue types.

**Fig 1:**
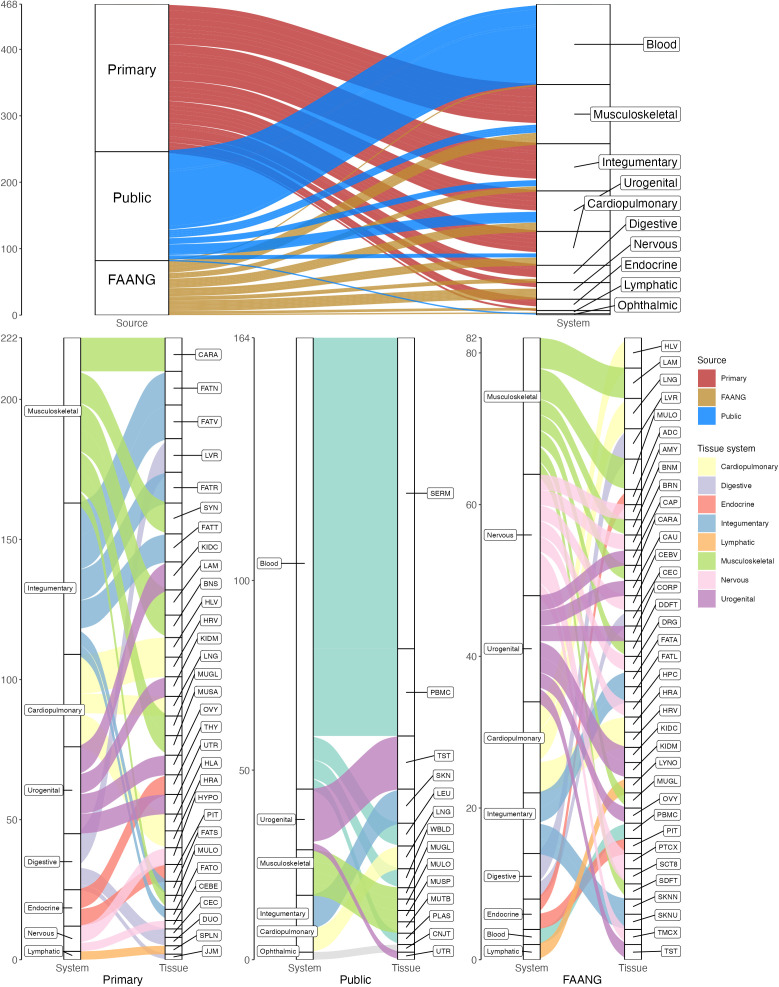
Tissue samples included for quantification by data source and system (top) and tissue type (bottom).

Following pre-processing, 28 samples (7 *primary*, 1 *FAANG*, and 20 *public*) were excluded due to post-filtered log_2_ read counts less than three median absolute deviations (MAD) from the median of all post-filtered log_2_ read counts (15 serum, 3 uterine fluid, 2 uterus, 2 longissimus muscle, and one each of lamina, left atrium, pituitary, retroperitoneal adipose, sesamoid bone, and subchondral bone). An additional 4 samples (1 *primary* and 3 *FAANG*) passed the MAD filter but consisted of <10% post-filtered reads assigned to miRNA biotypes (2 third metacarpal bone, 1 sesamoid bone, and 1 omental adipose). Subsequently, 3 tissue types were excluded as they had only one sample remaining (third metacarpal bone, sesamoid bone, and uterine fluid). This filtering reduced total tissue types from 64 to 61, with 3 tissue types having samples from all three sources (lung, gluteal and longissimus muscles), 14 from two, and 44 from a single source. Overall, the samples that were retained for quantification consisted of 222 (47%) *primary*, 164 (35%) *public*, and 82 (18%) *FAANG* (Fig 1). Most tissue types from *primary* data were musculoskeletal (59/222, 26.6%), integumentary (54/222, 24.3%), and cardiopulmonary (33/222, 14.9%). Musculoskeletal was also the predominant tissue type from *FAANG* (18/82, 22.0%), followed by nervous system (16/82, 19.5%) and urogenital (14/82, 17.1%). The *public* data were predominantly from blood and related tissues (119/164, 72.6%), followed by urogenital (16/164, 9.8%) and the remaining, constituting <8% per system.

**Fig 2:**
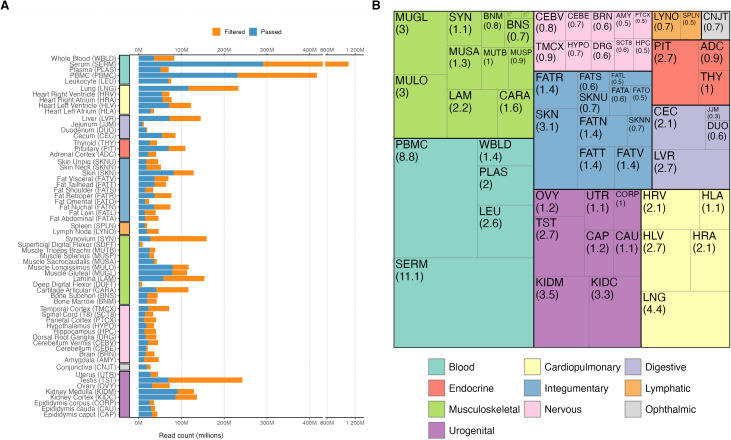
Total read counts of samples included for quantification. **(A)** Total passed and filtered read counts (in millions) across tissues. **(B)** Passed reads nested within the tissue system (percent of total reads in parentheses). (Note both deep digital flexor tendon (DDFT) and superficial digital flexor tendon (SDFT) have passed read percentages of 0.1).

After quality and length filtering, 2.6B post-filtered reads were retained with a median of 2.8 million (M) reads per tissue. Serum had the largest number of post-filtered reads (290.9M reads from 82 samples), followed by PBMC (231.5M reads from 25 samples) and lung (116.3M reads from 17 samples), with the smallest being deep digital flexor tendon (2.2M reads from 2 samples) (Fig 2A). Over half of all post-filtered reads are from only three tissue types: blood (25.9%), musculoskeletal (15.8%), and urogenital (15.0%) (Fig 2B).

**Fig 3:**
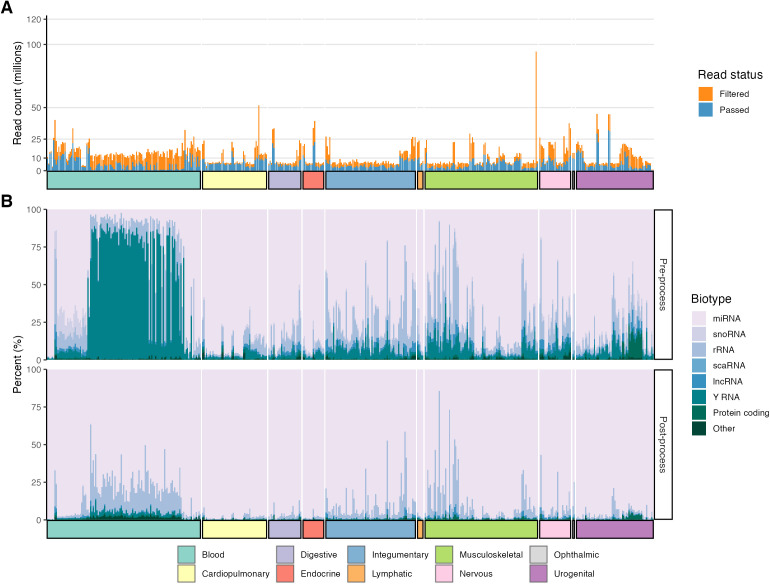
Per-sample passed and filtered read counts and biotype percentages pre- and post-processing. **(A)** Passed and filtered read counts (in millions) for each sample. **(B)** Percentage of biotypes per *featureCounts* pre- and post-processing by FARmiR.

Among the 468 tissue samples included for quantification, the median number of post-filtered reads was 4.4M (range 428,998–32,121,885) (S2 Fig). Sample-level post-filtered reads (Passed vs Filtered), and transcript type percentages for pre-process (i.e., adapter removed, simple quality filtered, and joined reads) and post-filtered (i.e., pre-quantification reads) are depicted in Fig 3A and 3B. Y RNA and rRNA were the predominant transcript types in 14.3% (67/468) and 5.6% (26/468) of pre-processed samples, respectively. Following processing, only 0.85% (4/468) of samples were dominated by rRNA (2 articular cartilage, 1 serum, and 1 skin) and 0 by Y RNA. Overall, the processing and removal of non-miRNA reads resulted in increased proportions of miRNA reads with a sample mean of 90.4% (SD = 11.9%) post-process compared to the pre-process mean of 66.5%, SD = 29.3%). These results suggest FARmiR processing was reliable in enriching for miRNAs.

Seven samples (6 serum and 1 kidney cortex) with a high proportion of missingness (i.e., at least 90% of miRNAs had RPMs of zero) were removed. The excluded kidney cortex exhibited a much higher percent reduction in passed reads (91.2%) compared to other kidney cortex samples (mean = 35.8%, SD = 10.1). All 6 of the excluded serum samples originated from the same BioProject and may reflect technical differences in library preparation or sequencing. The final expression set contained 461 samples from 61 tissue types. There were 299 tissues of female origin (7 fillies and 292 mares) and 162 of male (9 colts, 42 geldings, 70 stallions, and 41 unknown). Consequently, the total number of passed reads from female samples (1.54B) was 49.4% more compared male samples (1.02B) (S4 Table). However, when averaged per sample, we observed no statistical difference in passed read depth between female and male samples (mean difference = 40,046; 95% confidence interval (CI): -1.3M – 1.4M). The 461 samples span 15 breeds (plus 4 mixed parentage breeds) with 32.1% (823.3M) of the total passed read depth from Thoroughbreds (n = 83; excluding 2 mixed-breed samples), followed by Quarter Horse (n = 209; excluding 3 mixed-breed samples) with 31.8% (817.5M), and 13.0% (334.2M) from Warmbloods (n = 82) (S5 Table). Although there are imbalances in sample representation between sex and breeds, the consistent per sample read depth between sexes and breadth of tissues across multiple breeds provide a reasonable foundation for cataloging tissue-specific expression.

#### EquCab3 miRNA annotation and predicted novel miRNA loci.

**Fig 4:**
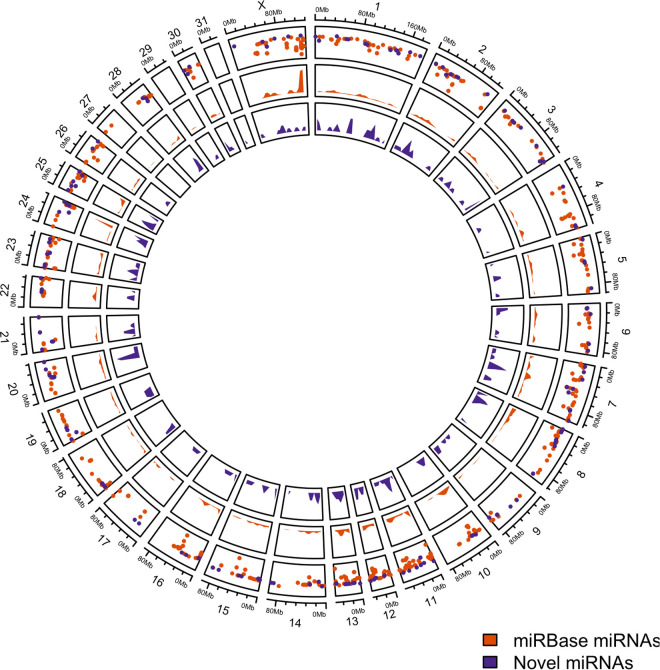
Genomic map of miRNA hairpin loci. Distribution of known (orange) and predicted (purple) miRNA hairpin loci on EquCab3. The outermost ring indicates loci density, with the Y-axis (log scale) representing the distance between neighboring loci.

Of the 715 hairpin loci lifted from EquCab2 to EquCab3, 24 were on unplaced contigs. To evaluate the updated miRNA annotation, only hairpins successfully lifted between the two reference genomes and located on chromosomes were considered (n = 673). The majority of hairpins were on the same chromosomes in both EquCab2 and the updated EquCab3 annotation (S3 Fig). From the 461 included samples, 217 mature miRNAs from 195 hairpin loci were predicted (S6 Table). Only 14 (7.2%) of the predicted hairpins were found in another species, compared to 150 (69.1%) of the mature miRNAs with seed matches to other species. A genomic map of canonical and predicted hairpin locations on EquCab3 is depicted in Fig 4.

#### Quantification of Ref-miRs/isomiRs.

A total of 83,057 miRNAs were detected from the included samples. Importantly, the same miRNA sequence can arise from different loci or precursors*.* For example, *eca-miR-26a* may be processed from two distinct hairpin loci *eca-mir-26a* (chr6:76,295,576–76,295,660(-)) or *eca-mir-26a-2* (chr16:48,848,855–48,848,932(-)). Similarly, the sequence of an *eca-miR-378* isomiR is identical to four novel isomiRs: *eca-novel-miR-2043* (orthologous to the *Bos taurus* (bta) precursor *bta-mir-378d*), *eca-novel-miR-10819*, *eca-novel-miR-13829*, and *eca-novel-miR-15239*. As a result, it is not always possible to confidently assign each mature miRNA sequence to a specific source. Collapsing identical mature sequences across multiple loci resulted in 73,131 distinct miRNA sequences. However, all mature miRNA names and sequence identifiers were retained and are accessible through AIMEE, allowing users to explore potential precursor origins and genomic loci for each sequence.

To reduce the likelihood of false positives, total miRNAs were filtered to include only those with RPMs > 20 in at least 2 samples. The final expression atlas contained 6,951 miRNAs with 5,781 unique mature sequences, of which 318 represent Ref-miRs (248 canonical miRBase entries and 70 novel), 5,448 isomiRs (4,593 canonical and 855 novel), and 15 that are both canonical Ref-miR and novel isomiR. Four of the 248 canonical miRBase entries have identical mature sequences, thus of the 690 distinct miRBase entries, 252 were identified. Of these 252, only 5.2% (13/252) were originally discovered experimentally [47], and the remaining 94.8% (239/252) were predicted *in silico* [46]. Compared to the 387 unique (438 total) miRNA sequences from MirGeneDB v3.0 [48], there were 259 (66.9%) exact matches. Additionally, 2,150 sequences showed full-length containment (1,117 atlas miRNA sequences wholly contained within MirGeneDB and 1,033 MirGeneDB sequences wholly contained within the atlas) bringing the total overlap to 2,409 (41.7%).

**Fig 5:**
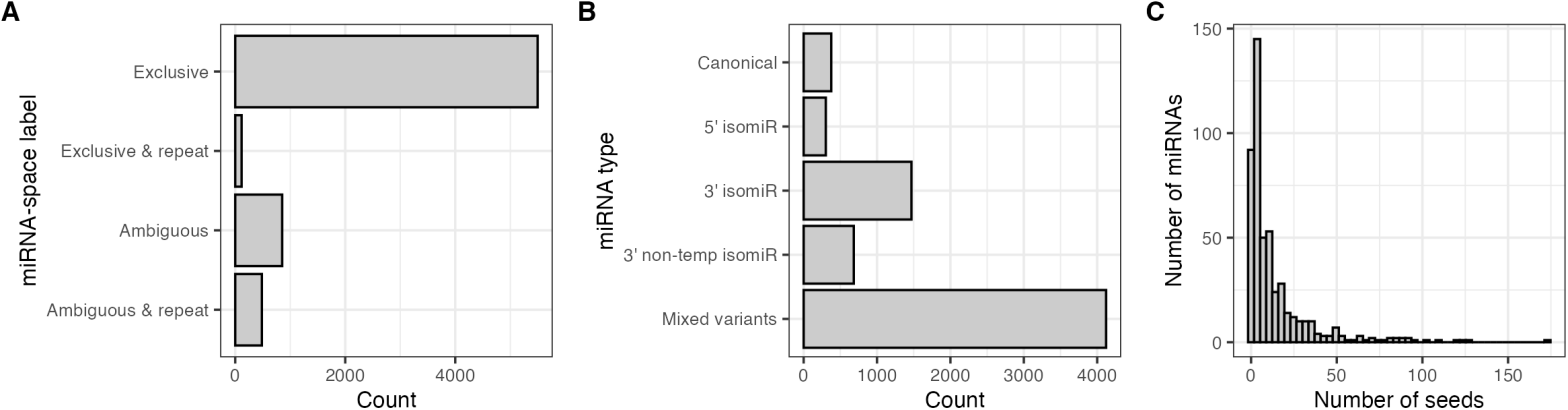
MiRNA location tags, variant types, and seed distribution. Counts of miRNA location labels **(A)**, canonical and isomiR forms **(B)**, and the distribution of seed shifting variant forms for each identified canonical and predicted Ref-miR **(C)**.

Quantification using *isoMiRmap* enables the ability to tag each identified miRNA sequence according to whether it is located exclusively within the “miRNA-space” (the union of all known and predicted hairpin sequences) [63]. In this way, miRNAs are tagged in reference to the miRNA-space as exclusive (found only within the miRNA-space) or ambiguous (found outside of the miRNA-space). Of the 6,951 miRNAs, 5,498 (79.1%) were exclusive to the miRNA-space, with an additional 116 (1.7%) also located within repeat islands (e.g., DNA, LINE, or SINE elements) (Fig 5A). The majority of identified miRNAs were isomiRs of “mixed” type (4,121/6,951 - 59.3%) (i.e., sequence changes at both the 5’ and 3’ ends compared to the canonical sequence), followed by strictly 3’ (1,469/6,951 - 21.1%), and 3’ non-templated (683/6,951 - 9.8%). IsomiRs containing only 5’ templated modifications were observed at the lowest frequency (302/6,951 - 4.3%) (Fig 5B). All identified isomiRs were derived from a total of 493 canonical and predicted Ref-miRs of which nearly half (237/493 - 48.1%) existed with at most five different seed regions (i.e., isomiRs with changes to the 5’ end) (Fig 5C). The top five miRNAs with the highest number of seed shift variants were *eca-miR-7* (n = 174), a novel miRNA *eca-novel-miR-6634* (n = 126), *eca-miR-140-3p* (n = 125), *eca-miR-1* (n = 121), and *eca-miR-133a* (n = 108).

**Fig 6:**
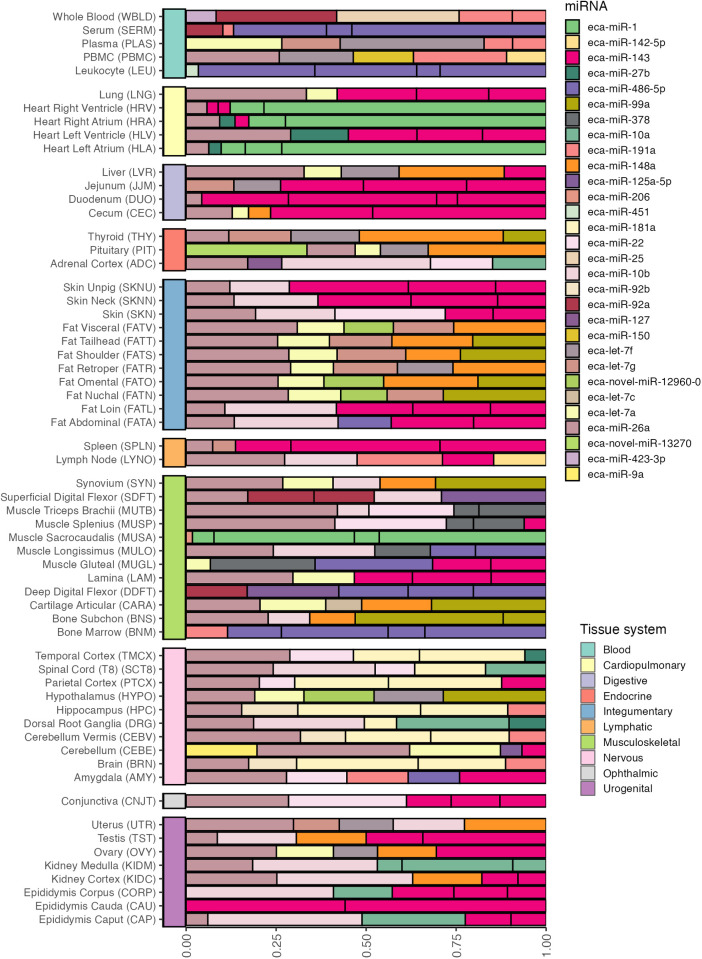
Proportions of the top 5 miRNAs per tissue based on robust rank aggregation.

Robust rank aggregation was used to identify the top five miRNAs per tissue across the entire atlas (Fig 6). The canonical form of *eca-miR-26a* was the most abundant, occurring in the top five for 50 of the 61 tissues (82.0%), followed by *eca-miR-143* in both canonical (24/61 - 39.3%) and 3’ templated (20/61 - 32.8%) forms, canonical *eca-let-7a* (18/61 - 29.5%), and *eca-miR-148a* (16/61 - 26.2%). Besides the canonical Ref-miR and isomiRs, two predicted miRNAs (named via the default *mirPRo* naming convention) were also ranked highly in select tissue types. Notably, the canonical form of the predicted miRNA *eca-novel-miR-12960–0* (chr6:80,187,888–80,187,910(+)) was in the top five for the hypothalamus and three adipose tissues (nuchal, omental, and visceral). Furthermore, a 3’ templated isomiR of *eca-novel-miR-13270* (chr6:8,343,131–8,343,150(-)) was in the top five across pituitary samples. Amongst the included tissues of the digestive tract (cecum, duodenum, and jejunum), either canonical or isomiR variants of *eca-miR-143* were top ranked. Similarly, in the epididymis cauda, *eca-miR-143* was overwhelmingly the most dominant miRNA. The canonical *eca-miR-1* was top ranked in the right ventricle of the heart and the left and right atria but not the left ventricle.

#### Tissue specificity.

**Fig 7:**
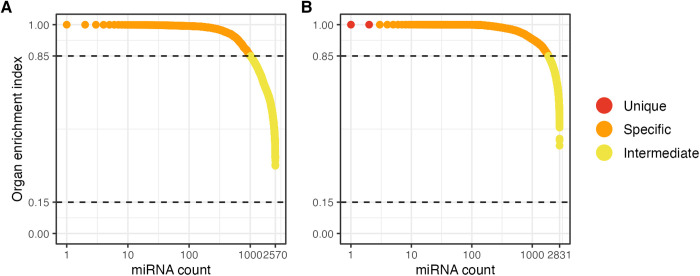
Organ enrichment index for miRNAs from *primary* (A) and *FAANG* (B) data.

Among the 5,781 unique mature miRNA sequences, 4,032 had a mean RPM > 20 in at least one organ across all included samples. Tissue specificity was determined using OEI values where OEI < 0.15 was considered ubiquitous, OEI between 0.15 and 0.85 intermediate, and OEI > 0.85 tissue-specific. As described in the methods, mean RPMs were calculated for each miRNA per organ. Tissue types from the same organ (skin sections, chambers of the heart, kidney regions, and brain subregions) were averaged together, while other tissues were retained separately. Only 9 (0.22%) miRNAs were detected in a single organ, 6 (0.15%) in two, 10 (0.25%) in three, while the remaining were observed in all (762/4,032 - 18.9%) or found in some but absent in others. Due to the unbalanced nature of the included projects and the resulting inability to control for technical variation between samples of the same tissue, OEIs were calculated separately for the *primary* (n = 2,570 miRNAs with at least one organ RPM mean > 20) and *FAANG* (n = 2,831) sources. Over half (1,560/2,570 - 60.7%) of miRNAs from primary were expressed within the intermediate OEI range (0.15 - 0.85), and 39.3% (1,010/2,570) were classified as tissue-specific (OEI > 0.85) (Fig 7A). Conversely, mean organ expression from FAANG samples yielded 34.5% (977/2,831) intermediate and 65.4% (1,852/2,831) tissue-specific miRNAs (Fig 7B). Moreover, two miRNAs were uniquely enriched (OEI = 1) in single tissues, a 3’-isomiR of *eca-miR-7* in the pituitary and a 5’-isomiR of *eca-miR-215* in the cecum.

### AIMEE usage examples

**Fig 8:**
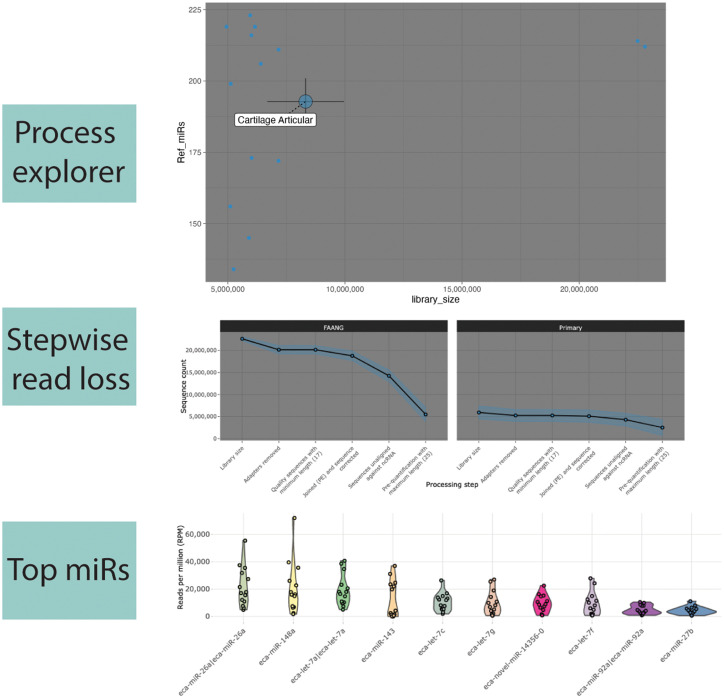
Select exploration module examples of the equine miRNA expression atlas supported by AIMEE. *Process explorer* (top) enables quick access to the counts of each processing step. In this example, the number of unique Ref-miRs is plotted as a function of library size for articular cartilage samples. Each point represents a single sample, and the larger points are the centroids of the highlighted tissue. *Stepwise read loss* (middle) charts the read attrition from library size through pre-quantification, which can be summarized at the tissue level (as shown) or displayed for each sample. *Top miRs* (bottom) allows visualization of the top N Ref-miRs or isomiRs based on mean RPMs from a selected source and tissue.

AIMEE delivers a range of analyses and visualizations for the processed miRNA expression data (Fig 8). We present two brief application scenarios an end user may undertake.

### Use case: select a tissue of interest, determine the top-expressed miRNAs, and explore their expression across all available tissues

**Fig 9:**
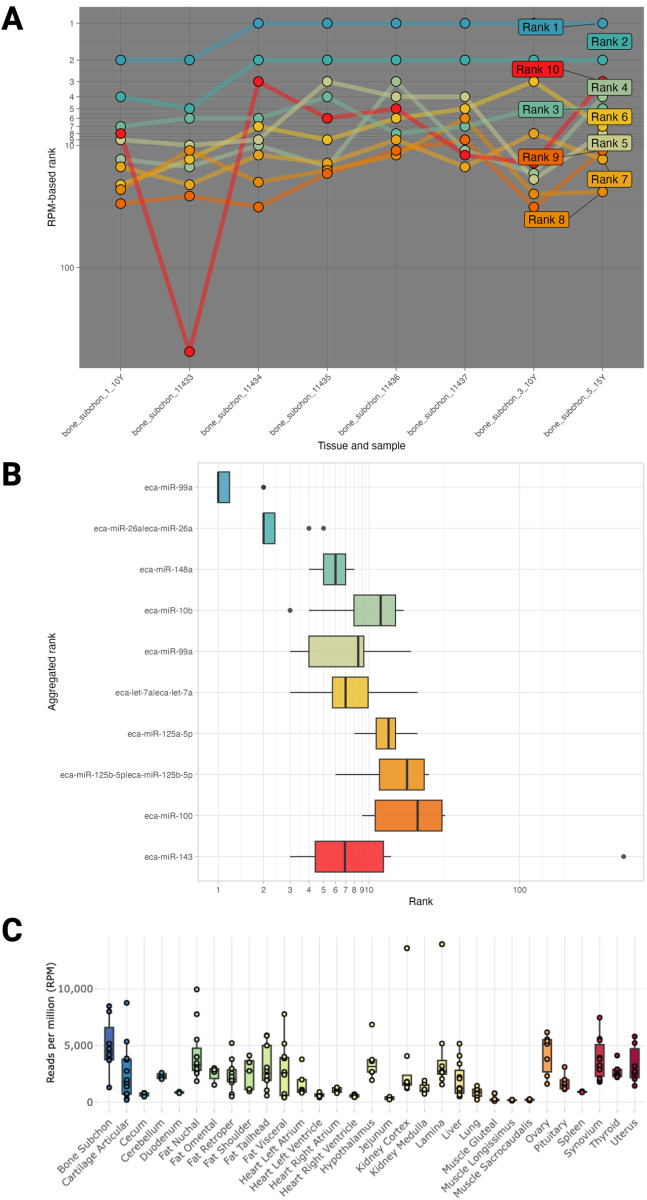
Application of AIMEE to identify top-ranked miRNAs in a tissue of interest. **(A)** Rank bump chart following robust rank aggregation from subchondral bone samples (n = 8). **(B)** Aggregated ranks of the top 10 expressed miRNAs. **(C)** Expression of the subchondral bone top ranked miRNA (*eca-miR-99a*) across all *primary* tissues.

Researchers are often interested in characterizing the miRNA profile from a specific tissue and identifying miRNAs that are consistently highly expressed within that tissue (e.g., across samples or time points). Without access to healthy tissue samples from which to prepare libraries and sequences, especially in the case of tissues requiring euthanasia, a researcher may need to either identify relevant data from public repositories or parse expression matrices and metadata. In both cases, advanced bioinformatics expertise is often required, and for the latter scenario, assumes the methodology used to generate the expression data was precise. AIMEE bypasses these hurdles, providing access without requiring exhaustive searches of public repositories for appropriate datasets or extensive bioinformatics experience. For example, the subchondral bone plays an important role in the development and progression of osteoarthritis (OA) in both horses and humans [64]. While there have been studies investigating miRNAs as potential biomarkers for OA in horses [53,65,66], subchondral bone has not yet been reported on in this context. Using the AIMEE *Rank aggregation* view, all available samples with subchondral bone data (n = 8; *primary* only) can be selected, and the top 10 miRNAs are calculated using RRA [67,68] and visualized using bump (Fig 9A) and box plots (Fig 9B). The top ranked miRNA, *eca-miR-99a*, can then be used as input to examine where else it is expressed (Fig 9C).

### Use case: exporting raw counts and differential expression

**Fig 10:**
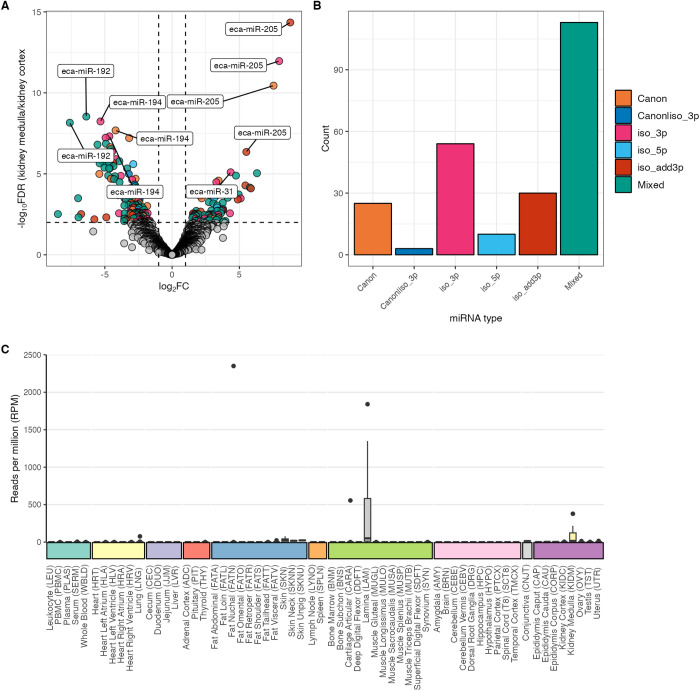
Application of AIMEE to export normalized expression data for downstream analysis. **(A)** Volcano plot of differentially expressed miRNAs between kidney medulla and cortex with log_2_ fold (X-axis) and -log_10_ FDR adjusted *p*-values. Grey circles represent non-significant miRNAs (FDR p-value > 0.01). The top five miRNAs with increased expression and top five miRNAs with decreased expression are labeled with the canonical name and colored by form. **(B)** Counts of the significant miRNA types from **A. (C)** Tissue-level RPM expression of *eca-miR-205* across the entire atlas.

Formatted raw (or normalized) expression data and metadata may be extracted. With careful consideration of the data source and potential batch effects, these exports are suitable for downstream analyses, including differential expression (DE) between tissues or samples of interest. To demonstrate this utility, un-normalized count and metadata data from kidney cortex and medulla samples were exported for DE analysis with DESeq2 [69]. Briefly, pre-filtering was applied to the un-normalized counts retaining only miRNAs with counts of at least 10 in a minimum of 6 samples (the smallest group size). Un-normalized (raw) counts of expressed miRNAs were analyzed using DESeq2 with a negative binomial generalized linear model that included individual horse and tissue type. Including horse or sample in the model accounts for the differences between individuals while estimating the effect due to tissue type. The Benjamini-Hochberg method was used to control the FDR with an adjusted *p*-value < 0.01 set for miRNAs to be considered differentially expressed. This more stringent threshold was chosen to avoid identifying false positives at the expense of false negatives. Kidney was chosen as an example as there are six paired samples per group or structure, the recommended minimum for DESeq2 DE [70], from the same source (*primary*) to minimize batch effects. Of the 2,673 pre-filtered non-zero miRNAs, 81 (3.0%) were significantly up-regulated (FDR *p*-value < 0.01), and 154 (5.8%) down-regulated in the medulla compared to the cortex (Fig 10A). Of the 235 DE miRNAs, 10.6% (10/235) were classified as canonical or predicted Ref-miRs, while 89.4% (210/235) were isomiRs. Moreover, when considering mixed type with 5’ modifications and 5’ isomiRs, 32.0% (57/235) of DE miRNAs had seed-shifting alterations (Fig 10B). The Ref-miR and IsomiRs of *eca-miR-192* were the most abundant DE miRNAs at 12.8% (30/235), all of which were down-regulated in the cortex compared to the medulla. A 3’ non-templated isomiR of *eca-miR-205* had the highest log_2_ fold change at 8.78 (FDR *p*-value < 0.001), which can be visualized as user-selected input with AIMEE (Fig 10C). Throughout the atlas, *eca-miR-205* expression was not restricted to the kidneys but was also observed in other tissues (e.g., lamina).

## Discussion

From 462 samples across 61 tissue types, AIMEE includes the largest collection of uniformly processed equine miRNA expression data to date. This expression atlas and explorer contains nearly 6,000 putative and canonical miRNA sequences, ~ 95% of which are isomiRs. The inclusion of isomiRs is of particular value as the importance of these molecules has rapidly grown in the last decade [28]. Consistent with previous studies, the equine atlas contains far fewer 5’-isomiRs compared to 3’-isomiRs [32]. Due to the well-known batch effects inherent in RNA-seq data [71,72], robust rank aggregation was used to circumvent the challenges associated with batch correction methods and integrate samples from heterogeneous sources. Also, in the context of minimizing unwanted technical variation, the tissue specificity index (TSI) was calculated for each unique miRNA that was sufficiently expressed (i.e., mean RPM > 20 in at least one organ) independently for the included *primary* and *FAANG* samples. From the *FAANG* samples, for example, a 3’-isomiR of *eca-miR-7* and a 5’-isomiR of *eca-miR-215* were exclusively expressed in the pituitary and cecum, respectively. The *miR-7* miRNA family has been associated with regulating gene expression in the pituitary of mice [73], rats [74], and cattle [75]. Similarly, the *miR-215* family has been repeatedly identified as a critical tumor suppressor in gastric and colorectal cancer [76,77], as well as associated with ulcerative colitis [78].

The scope of this miRNA expression atlas has implications for both basic research and future clinical applications. The tissue-specific miRNA and isomiR profiles generated as part of this work advances our understanding of gene regulatory mechanisms across equine tissues. In particular, the isomiR cataloging of various muscle types, cartilage, and blood, creates opportunities for biomarker discovery, improving precision in the diagnosis and monitoring of various equine conditions. Data from previous studies investigating the role of miRNAs in equine osteochondritis and osteoarthritis [49,79] can be analyzed along with the uniformly processed tissue data from this study, possibly contributing to the identification of additional biomarkers for early osteoarthritis detection. Furthermore, understanding the distribution of specific miRNAs across tissues is essential for developing targeted therapeutic interventions, particularly for metabolic and inflammatory conditions where miRNA-mediated regulation plays a significant role [80]. The characterization of isomiRs in individual tissues highlights new possibilities for personalized medicine approaches. For example, the isomiR catalog we developed for miRNAs like *eca-miR-7* (174 seed variants) and *eca-miR-140-3p* (125 seed variants) underscores the potential for individual variation in isomiR expression. This type of fine-tuned control of the regulatory machinery could have significant functional consequences that may warrant careful consideration in therapeutic strategies.

This equine miRNA expression resource does have some limitations. First, publicly available samples that met the inclusion criteria were based entirely on the descriptions reported by the authors in the original published main text, supplemental materials, or data repositories. Consequently, we cannot be as confident in the health assessments of these *public* samples compared to the *primary* or *FAANG* sets which included extensive ante and postmortem phenotyping. Second, we only identified 252 of the 690 miRBase-defined canonical miRNAs [25]. This is in contrast to previous studies that identified greater proportions, for example, in the testes [50,81]. This could be for a couple of reasons. Identifying miRNAs in earlier studies, especially in studies that were not using tools designed for miRNA identification but instead repurposing general aligners like BWA [82], can over-inflate miRNA counts. Even when using tools designed for miRNA profiling, there are significant differences in sensitivity and accuracy and poorly overlapping miRNA sets between tools [83]. Additionally, misannotated or spurious miRNAs in the reference set may hinder accurate identification and quantification. Many profiling tools utilize miRBase [25] or miRCarta [84] to define the miRNA space of a given species. For the horse, this may be suboptimal, as the 690 miRBase reference is based on two studies, with over half (359) being based on *in silico* prediction [46], and the remainder (331) from small RNA of testes of one horse of unknown breed and health status [47]. Both of these studies relied on the previous horse assembly [85]. In contrast, MirGeneDB v3.0 provides a curated set of 438 mature miRNAs (387 unique sequences) based on the current EquCab3 assembly [86]. We observed 259 of the 387 unique sequences (66.9%) exactly matched the mature sequences identified in this atlas, with an additional 2,150 exhibiting full-length containment. These findings underscore the potential biological complexity introduced by isomiRs and the challenges of maintaining comprehensive miRNA annotations. Importantly, FARmiR was not designed for this task but instead to catalog expression using the known miRNA space and predict additional miRNA loci beyond that space. As a result, it is possible that the parental miRNA precursors (and locations) have not been filtered against the current repeat element annotations, leading to hairpin-shaped precursors that are not true miRNAs [63]. Third, we did not include any *in silico* miRNA target prediction nor enrichment analyses based on predicted targets. This decision was made for two reasons: 1) it has been previously reported that computational target prediction (without experimental validation) in non-model organisms is not reliable [87]. 2) A follow-up to this study will couple prediction and validation to predict the RNA targets more accurately. Finally, this atlas profiled miRNA expression at the tissue-level, using bulk small RNA-seq. Characterization at the cellular level has demonstrated cell-specific expression of both human Ref-miRs and isomiRs [38,88,89]. Although still in its infancy and technically challenging, recent advances in HTS technologies have demonstrated the feasibility and potential for single-cell miRNA-seq protocols [90,91]. To date, there have been no rigorous characterizations of the equine miRNA landscape using single-cell RNA-seq methodology, thus representing a natural extension of this current work.

Using FARmiR and AIMEE represents a unified approach to miRNA data processing, analysis, and visualization. As an open-source, containerized pipeline for miRNA identification and quantification, FARmiR aligns with the principles of sustainable data analysis, promoting transparency, adaptability to other species, and *in silico* reproducibility [92,93]. AIMEE provides researchers a user-friendly and accessible option for exploring miRNA tissue profiles without the substantial effort and cost associated with preparing small RNA-seq data. AIMEE also includes a filtering option to consider only the mature sequences from this atlas that overlap with the curated set provided by MirGeneDB v3.0 [48]. The *primary*, *FAANG*, and *public* data, uniformly processed with FARmiR and accessible via AIMEE, contribute valuable information about the expression of equine miRNAs and, ultimately, the tissue-specific regulatory functions contributing to equine health and performance.

## Materials and methods

### Ethics statement.

Tissues were collected from horses donated for use in this study at the time of euthanasia. All procedures were approved by the Institutional Animal Care and Use Committee at the University of Minnesota (1712-35369A). All methods were performed in accordance with the IACUC guidelines and regulations.

Three small RNA-seq data sources were utilized for this atlas, one *primary* (i.e., the 12 horses enrolled as part of this study) and two public sources. The latter two sources consisted of four Thoroughbreds from the publicly available Functional Annotation of Animal Genomes (FAANG) consortium (referred to as *FAANG*), and systematic identification of compatible datasets from public databases.

### Primary data source

#### Horses.

A total of 12 healthy horses (11 American Quarter Horses and 1 American Paint Horse) donated to the University of Minnesota provided the *primary* source of data for this study. Physical examinations were performed by a boarded large animal internal medicine specialist to ensure horses were clinically normal. Horses were housed on a dry lot as a group and were fed free choice hay and water. Isolation and twice daily temperature checks were performed for two weeks to safeguard against communicable diseases. The *primary* source consisted of two cohorts with the first in the fall of 2013 (3 mares, one gelding, and one stallion 5–15 years of age) and the second in the fall of 2020 (7 mares 12–14 years of age).

#### Tissue collection and RNA isolation.

Horses were sedated with 0.05 mg/kg intravenous (IV) injection of xylazine, an alpha-2 adrenergic agonist, and humanely euthanized with ≥ 100 mg/kg injection of pentobarbital sodium (Fatal-Plus, Vortech Pharmaceutical) following the American Association of Equine Practitioners Euthanasia Guidelines (https://aaep.org/resource/euthanasia-guidelines/). Immediately following euthanasia, all tissues were collected in triplicate and flash frozen in liquid nitrogen or placed in RNAlater Stabilization Solution (ThermoFisher Scientific). Both flash-frozen and RNAlater aliquots were stored at -80°C until further processing. A total of 31 tissues were collected from the first cohort and up to 70 tissues from the second cohort (S1 Table). All tissues were collected within 45 min of euthanasia. The following tissues (n = 31) were selected for further processing: articular cartilage, lamina, subchondral bone, synovium, multiple muscles (gluteal, longissimus, and sacrocaudalis dorsalis), duodenum, jejunum, cecum, liver, spleen, thyroid, kidney (cortex and medulla), pituitary, cerebellum, hypothalamus, lung, heart (left and right atrium and ventricle), multiple fat depots (nuchal, omental, retroperitoneal, shoulder, tailhead, and visceral), ovary, and uterus. Only tissues with at least two biological replicates were included for library preparation and sequencing. Tissues were selected to capture a range of potential expression diversity across tissue types, as well as to represent those actively being investigated by our laboratory and others. The remaining tissues are biobanked in the University of Minnesota Equine Genetics and Genomics Laboratory tissue repository. RNA was isolated using tissue-type specific protocols to maximize RNA quality and yield. Due to wide diversity of tissue types and composition, no single extraction protocol was appropriate for all sampled tissues. Tissue type specific protocols were developed and refined based on composition and assessment of RNA quality via NanoDrop 8000 spectrophotometer (Thermo Scientific, cat. no ND-8000-GL, Waltham, MA, USA) and the Agilent TapeStation system (Agilent Technologies, Santa Clara, CA, USA). See Supplemental Materials for complete descriptions of isolation protocols. Tissues isolated and sequenced as part of this study are referred to as *primary* throughout the remainder of this manuscript.

#### Small RNA library preparation and sequencing.

Sample QC, library preparations, and sequencing reactions were conducted at GENEWIZ, LLC/Azenta US, Inc (South Plainfield, NJ, USA). The RNA samples were quantified using a Qubit 2.0 Fluorometer (ThermoFisher Scientific, Waltham, MA, USA), and RNA integrity (RIN) was checked using the Agilent TapeStation (Agilent Technologies, Santa Clara, CA, USA). The Small RNA sequencing libraries were prepared using the NEBNext Small RNA Library Prep Kit for Illumina using the manufacturer’s instructions (New England Biolabs, Ipswich, MA, USA). Briefly, Illumina 3’ and 5’ adapters were added to RNA molecules with a 5’-phosphate and a 3’-hydroxyl group sequentially. A reverse transcription reaction was used to create single-stranded cDNA. The cDNA was then PCR amplified using a common primer and a primer containing an index sequence. The amplified cDNA construct was purified by polyacrylamide gel electrophoresis, and the correct band (~145–160 bp) was excised from the gel and eluted with water. The eluted cDNA was concentrated by EtOH precipitation generating the sequencing libraries. The sequencing libraries were multiplexed and clustered onto a flowcell. After clustering, the flowcell was loaded onto the Illumina HiSeq 4000 or NovaSeq 6000 instrument according to the manufacturer’s instructions. The samples were sequenced using a 2 × 150 bp paired-end (PE) configuration. Image analysis and base calling were conducted by the HiSeq Control Software (HCS). Raw sequence data (.bcl files) were converted into FASTQ files and de-multiplexed using Illumina bcl2fastq 2.20 software. One mismatch was allowed for index sequence identification.

### Equine Functional Annotation of ANimal Genomes (FAANG) data

#### Ethics statement.

All procedures were approved by the Institutional Animal Care and Use Committee at the University of California - Davis (protocols #19037 and 21033). All methods were performed in accordance with the IACUC guidelines and regulations.

#### Horses.

As part of the equine FAANG project, small RNA-seq libraries were constructed and sequenced using tissue samples collected from two healthy Thoroughbred mares (aged 4 and 5 years) [61] and two healthy Thoroughbred stallions (aged 3 and 4 years) [62].

#### Tissue collection and RNA isolation.

Horses were sedated and humanely euthanized prior to sample collection and preservation as described [61,62]. The following tissues (n = 39) were selected for RNA isolation and miRNA sequencing: adrenal cortex, amygdala, articular cartilage (fetlock and stifle), bone marrow, brain, cecum, cerebellum vermis, deep digital flexor tendon, dorsal root ganglia, epididymis (caput, cauda, and corpus), fat depots (abdominal and loin), heart (left and right ventricle, right atrium), hippocampus, kidney (cortex and medulla), lamina, liver, lung, lymph node, muscle types (gluteal and longissimus dorsi), ovary, parietal cortex, peripheral blood mononuclear cells (PBMC), pituitary, sesamoid bone, two skin sections (neck and unpigmented), superficial digital flexor tendon, T8 thoracic vertebrae, temporal cortex, testis, and third metacarpal bone (S2 Table).

#### Small RNA library preparation and sequencing.

Sample QC, library preparations, and sequencing reactions were conducted at the University of Minnesota Genomics Center (UMGC) (n = 40) or Admera Health (n = 46). At UMGC, total RNA isolates were quantified using a fluorimetric RiboGreen assay on a BioTek Synergy 2 (BioTek Instruments, Winooski, VT, USA). Total RNA integrity was assessed using capillary electrophoresis (Agilent BioAnalyzer 2100, Agilent Technologies, Santa Clara, CA, USA) to generate RNA Integrity Numbers (RIN). While UMGC recommends total RNA yields of at least 500ng and RINs > 8, TruSeq Small RNA libraries were prepared for samples (n = 36) with a range of RINs (5.4 – 9.5). Libraries for four bone-derived samples (two sesamoid and two third metacarpals) with the lowest RINs (3.7 – 4.0) were prepared using the Clontech smRNA-Seq kit (Takara Bio USA, San Jose, CA, USA) for Illumina following the manufacturer’s instructions, which is more accommodating for low-input RNA. The sequencing libraries were multiplexed and clustered onto a flowcell. After clustering, the flowcell was loaded onto the Illumina HiSeq 2500 and sequenced using a 2 × 50 bp PE configuration. Raw sequence data (.bcl files) were de-multiplexed and converted into FASTQ files using CASAVA v1.8.2 software. At Admera Health, isolated RNA sample quality was assessed by BioAnalyzer RNA Eukaryotic Nano Assay (Agilent Technologies, Santa Clara, CA, USA) and quantified by Invitrogen Broad Range RNA Qubit Assay (Invitrogen, Thermo Fisher Scientific, Waltham, MA, USA). Library construction was performed with TruSeq Small RNA kit (Illumina, San Diego, CA, USA). Final libraries quantity was assessed by Qubit 2.0 Fluorometer (ThermoFisher Scientific, Waltham, MA, USA) and quality was assessed by TapeStation HSD1000 ScreenTape (Agilent Technologies, Santa Clara, CA, USA). Mean final library size was about 200 bp with an insert size of about 50 bp. Illumina 6-nt single-indices were used. Equimolar pooling of libraries was performed based on QC values and sequenced on an Illumina NovaSeq 6000 with a read length configuration of 150 PE for 20M PE reads per sample (10M in each direction) and data was trimmed to 1x50 sequencing configuration. Tissue samples sequenced across different runs or lanes were combined prior to processing.

In the *primary dataset,* both fragment ends were sequenced for all libraries. However, only the first read of the *FAANG* data was available and the included *public* samples were sequenced from single-end (SE) libraries. Therefore, only the first read from the *primary* dataset was included to mitigate library configuration as a source of unwanted technical variation or batch effect, an unavoidable and inevitable challenge with aggregating multi-center RNA-seq data [71,94].

### Public data sources

#### Database search and validation.

The National Center for Biotechnology Information (NCBI) BioProject and PubMed databases were queried using “(equine OR horse) AND (miRNA OR microRNA)” on April 4, 2023, and May 3, 2023, respectively, to identify potentially relevant studies and datasets. The search was limited to studies and datasets describing Illumina-based small RNA-seq of equine tissue samples with publicly available sequencing data. Studies that profiled non-blood derived fluids/cell types (e.g., semen, milk, synovial fluid), extracellular vesicles, or other culture-expanded cell types (e.g., skeletal muscle satellite cells, chondrocytes) were excluded to focus on native tissue-derived expression. These sample types were omitted due to the potential for increased technical variability associated with the additional, type-specific handling and preparation steps, the evaluation of which was beyond the scope of this study. In contrast, blood derived sample types were included due to their widespread usage and established relevance in miRNA expression studies despite variability in processing. From each study, samples were considered eligible if described as healthy without experimental treatment. For example, data from the pre-challenge samples in a challenge study [95] were included, while data from horses described as “non-performers” in an exercise or endurance trial were excluded [56]. Similarly, if horses were not described as “non-performers,” both pre- and post-race samples were considered eligible [55]. Samples were excluded if health status was not described or could not be inferred from available metadata. Since it has been suggested that sex and breed-specific miRNA profiles may exist in the horse [58] and other species [96,97], samples lacking sex or breed information were also omitted. Mixed breeds were only included if at least one parental breed was described (i.e., horses labeled “cross” were excluded, whereas horses labeled “Thoroughbred-cross” were included). Samples described as male were classified as male-unknown unless expressly indicated as stallion or gelding. In the case of females, if age was reported, a distinction was made between mare (>3 years) and filly. Similarly, males under 3 years were labelled colt. All sample data passing inclusion criteria were downloaded from the NCBI Sequence Read Archive (SRA) with *parallel-fastq-dump* v0.6.6 (https://github.com/rvalieris/parallel-fastq-dump) (a *fastq-dump* wrapper from the SRA toolkit v2.11.0 [98]) or directly from the European Nucleotide Archive (ENA). Study details and sample data for included *public* samples are reported in S3 Table.

### Small RNA processing and analysis

**Fig 11:**
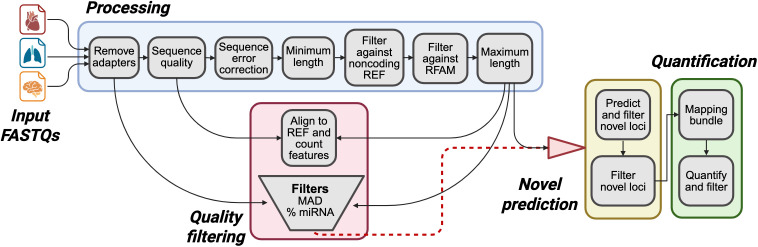
Overview of the FARmiR pipeline. Created with BioRender.com.

We developed the Framework for Analysis and Refinement of miRNAs (FARmiR; https://github.com/jonahcullen/FARmiR) pipeline to catalog miRNA expression in a reproducible, transparent, and adaptable manner, adhering to the principles of sustainable data analysis [92]. FARmiR is an open-source, Snakemake-based [92] containerized pipeline [93] developed for quality assessment, pre-processing, and miRNA profiling (Fig 11). Although designed for use with any animal species, we illustrate its application and inputs using the current equine reference genome EquCab3 [86].

#### Read quality assessment and pre-processing.

Raw sequence reads from all three sources were first assessed for quality without any filtering using *fastp* v1.3.1 [99]. Adapters and low-quality reads (> 40% of bases with phred quality scores < 15 or at most 5 missing base calls) were removed. For SE samples, the initial approach employs *adapt_find* to identify and remove adapters [100]. If adapters were not detected, *DNApi* is used for adapter prediction followed by *cutadapt* [101,102]. Although not included here, adapter detection and removal with *fastp* is used for samples with PE layouts [99]. The adapter prediction functionality of *fastp* was not included for SE samples due to observed inconsistencies in adapter prediction across datasets. We instead implemented this sequential approach to improve robustness without requiring prior knowledge of the adapter sequence, which is often not readily available in the publication. If included, PE reads will be joined (no mismatches allowed and a minimum overlap of six bases required) [103]. By convention, miRNA profiling typically only considers SE-sequenced libraries (or the first read of PE layouts). We designed FARmiR to handle PE layouts as there is recent evidence that PE sequencing of small RNA-seq libraries may improve isomiR identification and accuracy [104]. Sequence error correction, a critically important consideration for isomiR discovery and tissue-specific quantification, was conducted with *miREC* [105], followed by length filtering (minimum length of 17) [99]. To remove likely non-miRNA molecules, cleaned reads were first aligned against the EquCab3 [86] non-coding reference (excluding miRNA sequences) from Ensembl (release 103). Unmapped reads were then aligned against non-coding RNA (excluding miRNA sequences) from the Rfam v14.8 database [106]. Both alignments use *bowtie* v1.3.1 [107] with zero mismatches permitted (“-n 0”), only one reportable alignment allowed (“-m 1”), and without alignment against the reverse-complement (“--norc”). Reportable alignments were limited to one as there are existing miRNA sequences that have significant overlap with other non-miRNA regions, such as piRNAs [108]. Reads unmapped against Rfam with lengths greater than 25 bases were removed (referred to as *post-filtered reads*) [99].

Pre- and post-filtered reads were aligned against EquCab3 using *bowtie* v.1.3.1 [107]. Default parameters were used except allowing for no mismatches (“-n 0”) within the first 8 bases (“-l 8”), up to 6 multiple alignments (“-m 6”) and reporting only the best stratum alignment (“-a --best --strata”). A maximum of 6 multiple alignments were permitted as there are at most 6 multimaps between the miRBase mature and hairpin sequences. We note however there exist up to 11 multimaps between human mature and hairpin sequences. Mapped reads and the Ensembl (release 103) EquCab3 annotation were input to *featureCounts* v2.0.1 [109], allowing for multi-mapped reads (“-M”) with per-exon counting (“-t exon”). MultiQC was used to aggregate pre-processing steps [110].

#### Sample filtering.

Samples with log_2_ post-filtering counts less than three times the unbiased median absolute deviation were excluded [111]. Following this, samples with reads consisting of less than 10% miRNA biotypes (as estimated by *featureCounts*) were also discarded. Finally, only tissues with at least two independent samples (i.e., regardless of age, sex, or breed) were retained for quantification. This approach was taken to improve overall data quality and reduce the influence of batch effects.

#### Updated miRNA annotation.

The current version of the equine miRNA annotation from miRBase is based on EquCab2 [85], and to the best of our knowledge, an EquCab3 annotation is not publicly available. To address this, we used a combination of *Liftoff* [112] and *LiftOver* [113] to map hairpins and mature sequence loci from EquCab2 to EquCab3. Of the 715 non-redundant miRNA hairpin genes (4 hairpins are encoded at more than one locus: *eca-mir-703* at 5 loci, *eca-mir-9128–2* at 3, and both *eca-mir-1842* and *eca-mir-9150* at 2 loci each), 7 hairpins were unmapped during conversion to EquCab3. All 7 unmapped hairpin loci (*eca-mir-8921–1*, *eca-mir-8921–2*, *eca-mir-8923–4*, *eca-mir-8923–5*, *eca-mir-8934–1*, *eca-mir-8935–6*, and *eca-mir-8946*) were predicted to be on unplaced EquCab2 contigs and are not included in either the Ensembl 103 or most recent 109 annotations. However, of the 690 mature miRNA sequences in EquCab2 (59 miRNAs are encoded by more than one hairpin), only one (*eca-miR-8946*) was not identified in EquCab3.

### Discovery and quantification of miRNAs

#### Novel miRNA loci.

Samples that passed filtering were used for novel miRNA prediction with *mirPRo* v.1.1.4 [114]. *MirPRo* uses *Novoalign* v3.02.12 [115] and a prediction algorithm similar to *miRDeep2* [116] with modifications to reduce false positives. Novel prediction was performed using default parameters with mature and hairpin miRNA sequences of human, mouse, dog, cow, and pig from miRBase v22 as related species [25]. miRBase v21 family information was used as miRBase v22 does not contain miRNA family information. There is no apparent difference between miRBase v21 and v22 families for equine miRNAs. *BLAST+* v2.13.0 [117] was used to query predicted hairpins against equine RNA sequences from RNAcentral [118] to remove other possible hairpin-forming RNAs (e.g., tRNA and rRNA). Genomic loci of predicted hairpins with a maximum overlap of 17 (based on a minimum mature sequence length of 17 as above) were merged with *BEDtools* [119]. Predicted hairpin sequences were then queried against the EquCab3 reference genome, and sequences with at most 15 alignments were retained. Predicted hairpin counts were then normalized using the post-filter counts from above to reads per million (RPM) and filtered to include only hairpins with RPMs ≥ 20 in at least 2 samples. In the cases of overlapping and merged loci, the hairpin with the highest total counts across all samples was retained. If total counts were equal between two or more hairpins, one was randomly selected to define the genomic locus. Expressed candidate loci that overlap (maximum of 17) with the updated miRNA annotation hairpin loci were removed. Retained expressed candidate loci were then used to select the associated mature representative. Novel miRNAs were named following a modified strategy previously established for sheep miRNA profiling [120]. Briefly, retained candidate loci were queried (*BLAST+* v2.13.0 with default settings and -*task blastn*) against the miRBase precursor sequences for cattle, sheep, pig, mouse, and human. Alignments with a q-value (minimum false discovery rate (FDR)) below 0.01 and sufficient query coverage (> 80%) were retained and the subject(s) with the highest percent identity was selected. For queries with valid alignments to more than one species, the evolutionarily closest species was chosen for naming. Note the original *mirPRo* naming convention (i.e., *eca-novel-mir-NNNN*) was preserved for downstream analyses and within AIMEE to avoid potential confusion. Orthologs of predicted miRNAs are noted where applicable and available in the supplemental data (S4 Table). The candidate hairpins and mature miRNAs were converted to BED format and merged with the updated miRNA annotation to be utilized for quantification.

#### Ref-miRs and isomiRs.

Quantification was conducted with *isoMiRmap* [63]. *IsoMiRmap* requires species-specific “mapping bundles.” Currently, the developers only provide a human miRNA mapping bundle. To address this, we generated a species mapping bundle prior to quantification, starting with the putative miRNA annotation (see Loher et al. (2021) [63] for details and https://github.com/jonahcullen/FARmiR for implementation). The post-filtered and sized reads from each sample, along with the mapping bundle, were then quantified by *isoMiRmap*. This procedure returns (among other outputs) a Ref-miR/isomiR miRGFF3 annotation [121], a proposed standard from the miRNA TRanscriptomic Open Project (miRTOP), which utilizes a previously described naming convention [122] and nucleotide-based unique identifier per isomiR [123]. RPM (normalized by the final post-filtered and sized read counts) and count data were extracted from these annotations and assembled into an expression matrix. Ref-miR/isomiRs were retained only where at least 2 samples have RPMs greater than 20. Following this, samples consisting of less than 10% non-zero RPMs were excluded, and ultimately, tissues were dropped if less than 2 independent samples remained. While this filtering improves interpretability, it may impact detection of lowly expressed miRNAs, particularity in sample types with high compositional variability across RNA biotypes. Various metrics were then calculated for each Ref-miR (e.g., numbers of hairpins, isomiRs, and seed-shifted isomiRs).

#### Shiny app and browser.

To promote accessibility and maximize insights derived from the collected data, we developed a Shiny web application built with the *golem* framework [124]. This app, Animal IsomiR and MiRNA Expression Explorer (AIMEE; https://github.com/jonahcullen/AIMEE), was designed to provide user-friendly accessibility and point-and-click functionality for data exploration and visualization, simple analyses, and exporting quantification data for subsequent investigations. AIMEE utilizes expression matrices generated by FARmiR and associated sample metadata as input for multiple visualization and analysis modules. These include simple quality control, read loss through FARmiR processing, isolating Ref-miRs/isomiRs of interest, and upset analysis with *ComplexUpset* [125]. Assessing inter- or intra-tissue Ref-miR/isomiR rank aggregation and tissue-specificity via the organ enrichment index (OEI) is also possible with AIMEE.

#### Robust rank aggregation.

Unbiased integration of small RNA-seq data is critical when attempting to make inferences amidst unknown or unquantified sources of technical variation. One such approach to reduce these biases is to represent each sample as an independent prioritized miRNA gene list (i.e., miRNAs ranked by expression at a user-defined threshold). These gene lists can then be aggregated using order statistics, with *p*-values assigned to each miRNA denoting how its rank in each sample list surpasses what would be expected by chance. AIMEE implements the R package *RobustRankAggreg* (RRA) [67,68], a probabilistic aggregation model that is robust to the variations associated with high-throughput data from disparate origins, to assign significance probabilities to each miRNA from user-selected tissue or sample sets. As RRA operates on within-sample rankings as opposed to raw expression values, it helps reduce – but does not eliminate – the impact of technical variation across samples. A more traditional batch effect correction (e.g., ComBat from the *sva* R package [126]) was not feasible given the diverse and unbalanced nature of the included samples.

#### Organ-enrichment index.

The OEI is a measurement of miRNA specificity [14,127], analogous to the mRNA tissue specificity index (TSI) ‘tau’ [128]. OEI values range from 0 to 1, with scores close to 0 indicating a miRNA that is expressed at similar levels across many or most organs (i.e., ‘housekeepers’), and scores close to 1 corresponding to miRNAs expressed in only one specific organ (i.e., organ-enriched). To calculate OEI, tissue types originating from the same organ were grouped together. Specifically, heart chambers were collapsed into “heart”, kidney regions into “kidney”, and skin samples into “skin”. Brain regions (parietal and temporal cortex, hippocampus, hypothalamus, amygdala, cerebellum and cerebellum vermis, and dorsal root ganglia) were combined into a single “brain” grouping. All other tissues were retained as separate groupings to preserve potential site-specific expression and enrichment. The mean RPM was calculated for each miRNA per organ or combined organ grouping. Only miRNAs with a mean RPM > 20 in at least one organ or group were retained. Then, the OEI was calculated for each miRNA *j* as


$$oei_{j\;}=\frac{\sum_{i=1}^{N}\left(1-x_{j,i}\right)}{N-1}$$

where *x*_*j,i*_ represents miRNA *j* in organ *i* normalized by the maximum RPM across all organs, and *N* is the total number of organs considered. Importantly, due to the likely presence of batch effects, AIMEE contains OEI values calculated separately using the *primary* or *FAANG* collection.

## Supporting information

S1 Text The pdf file contains RNA extraction protocols based on biochemical composition (e.g., lipid-rich, protein-rich). (PDF)

S1 Fig Flow diagram of the search, screening, and selection of publicly available small RNA-seq datasets. Created with BioRender.com.
(TIFF)

S2 Fig Counts (in millions) of filtered and passed reads following processing with FARmiR. (TIFF)

S3 Fig Mapping hairpin loci between EquCab2 (blue) and EquCab3 (yellow). (TIFF)

S1 Table Sample information and RNA-seq metadata for *primary* data. (XLSX)

S2 Table Sample information and RNA-seq metadata for *FAANG data.* (XLSX)

S3 Table Sample information and RNA-seq metadata for *public* data. (XLSX)

S4 Table Post-processed total, mean, and standard deviation of passed read counts by sex per tissue for the final expression set. (XLSX)

S5 Table Post-processed total, mean, and standard deviation of passed read counts by breed for the final expression set. (XLSX)

S6 Table Description of novel equine miRNAs. (XLSX)
