# Supplementary material for: Charting the equine miRNA landscape: An integrated pipeline and browser for annotating, quantifying, and visualizing expression
Source: PLoS Genet. 2025 Sep 5;21(9):e1011835. doi: 10.1371/journal.pgen.1011835 (PMC12449019; doi:10.1371/journal.pgen.1011835)
Supplement: S1 Text — (PDF) [file pgen.1011835.s001.pdf]

Supplemental Materials

RNA isolation protocols

Nucleic acid and nuclease-rich tissues

Total RNA isolation was performed using a TRIzol Reagent-chloroform extraction, the Qiagen RNeasy Mini Kit (Qiagen, cat.no 74104), and the Qiagen RNAse-free DNAse Set (cat.no 79254). Approximately 30 - 50 mg of frozen tissue were used to isolate total RNA. Tissue homogenization was achieved using the BioPulverizer (BioSpec, cat.no 59012N) and 1 mL of TRIzol Reagent using the Tissue-Tearor homogenizer (BioSpec, model 985370-395).

The homogenized lysate was incubated for 5 minutes at room temperature. Afterward, the lysate underwent an organic extraction with 200 µl of chloroform. The lysate and chloroform mixture was incubated for 3 minutes. A centrifugation step at 11,340 RPM at 4℃ for 15 minutes was performed to obtain the aqueous phase. The remaining total RNA isolation of the aqueous phase was achieved using the Qiagen RNeasy Mini Kit manufacturer's protocol starting at Step 2 with an on-column DNAse treatment utilizing the Qiagen RNAse-free DNAse Set. The quality of the samples was assessed by NanoDrop 8000 spectrophotometer (Thermo Scientific, cat.no ND-8000-GL) and the Agilent TapeStation system.

Lipid rich tissues (the fats)

Total RNA isolation was performed using a QIAzol Lysis Reagent-chloroform extraction, the Qiagen RNeasy Mini Kit (Qiagen, cat.no 74104), and the Qiagen RNAse-free DNAse Set (cat.no 79254). Approximately 80 - 100 mg of frozen tissue were used to isolate total RNA. Tissue homogenization was achieved using the BioPulverizer (BioSpec, cat.no 59012N) and 1 mL of QIAzol Lysis Reagent using the Tissue-Tearor homogenizer (BioSpec, model 985370-395).

The homogenized lysate was incubated for 5 minutes at room temperature before undergoing a centrifugation step at 11,340 RPM at 4℃ for 10 minutes. Then, the oil layer was removed from the lysate, which was transferred to a new 2.0 mL microcentrifuge tube.

Organic extraction was performed by adding 200 µl of chloroform to the lysate. The lysate and chloroform mixture was incubated for 3 minutes. A second centrifugation step at 11,340 RPM at 4℃ for 15 minutes was performed to obtain the aqueous phase. The remaining total RNA isolation of the aqueous phase was achieved using the Qiagen RNeasy Mini Kit manufacturer's protocol starting at Step 2 with an on-column DNAse treatment utilizing the Qiagen RNAse-free DNAse Set. The quality of the samples was assessed by the NanoDrop 8000 spectrophotometer (Thermo Scientific, cat.no ND-8000-GL) and the Agilent TapeStation system.

Articular cartilage - Protein-rich tissues

Total RNA isolation was performed using a TRIzol Reagent-chloroform extraction, the Qiagen RNeasy Mini Kit (Qiagen, cat.no 74104), and the Qiagen RNAse-free DNAse Set (cat.no 79254). Approximately 60 - 80 mg of frozen tissue were used to isolate total RNA. Tissue homogenization was achieved using the BioPulverizer (BioSpec, cat.no 59012N) and 1 mL of TRIzol Reagent in the Precellys Lysing Kit tubes (cat. P000916-LYSKO-A) using the Tissue-Tearor homogenizer (BioSpec, model 985370-395).

The homogenized suspension was divided into four new 2.0 mL microcentrifuge tubes. Organic extraction was performed by adding 200 µl of chloroform to the lysate. The lysate and chloroform mixture was incubated for 3 minutes. A centrifugation step at 11,340 RPM at 4℃ for 15 minutes was performed to obtain the aqueous phase.

The remaining total RNA isolation of the aqueous phase was achieved using the Qiagen RNeasy Mini Kit manufacturer's protocol starting at Step 2 with an on-column DNAse treatment utilizing the Qiagen RNAse-free DNAse Set. The quality of the samples was assessed by the NanoDrop 8000 spectrophotometer (Thermo Scientific, cat.no ND-8000-GL) and the Agilent TapeStation system.

Subchondral bone - Protein-rich tissues

Total RNA isolation was performed using the Invitrogen Mirvana Kit (cat. AM1560), the Qiagen RNeasy Mini Kit's clean-up protocol (Qiagen, cat.no 74104), and the Qiagen RNAse-free DNAse Set (cat.no 79254). Approximately 100 mg of frozen tissue was used to isolate the total RNA. Tissue homogenization was achieved using the BioPulverizer (BioSpec, cat.no 59012N) and 1 mL of Lysis/Binding buffer in the Precellys Lysing Kit tubes (cat. P000916-LYSKO-A) using the Tissue-Tearor homogenizer (BioSpec, model 985370-395).

The remaining total RNA isolation of the homogenized lysate was achieved using the Invitrogen Mirvana Kit manufacturer's protocol. The total RNA was further purified using the Qiagen RNeasy Mini Kit manufacturer's clean-up protocol with an on-column DNAse treatment utilizing the Qiagen RNAse-free DNAse Set. The quality of the samples was assessed by the NanoDrop 8000 spectrophotometer (Thermo Scientific, cat.no ND-8000-GL) and the Agilent TapeStation system.

Fiber-rich tissues

Total RNA isolation was performed using the Invitrogen Mirvana Kit (cat. AM1560). Approximately 60 - 80 mg of frozen tissue were used to isolate total RNA. Tissue homogenization was achieved using the BioPulverizer (BioSpec, cat.no 59012N) and 600-800 µl of the kit's Lysis/Binding buffer using the Tissue-Tearor homogenizer (BioSpec, model 985370-395).

The remaining total RNA isolation of the homogenized lysate was achieved using the Invitrogen Mirvana Kit manufacturer's protocol. The total RNA was eluted in 100 µL of pre-heated (95°C) nuclease-free water. Assessment of the samples' quality was verified by the NanoDrop 8000 spectrophotometer (Thermo Scientific, cat.no ND-8000-GL) and the Agilent TapeStation system.
