# Supplementary figures and images for: Charting the equine miRNA landscape: An integrated pipeline and browser for annotating, quantifying, and visualizing expression
Source: PLoS Genet. 2025 Sep 5;21(9):e1011835. doi: 10.1371/journal.pgen.1011835 (PMC12449019; doi:10.1371/journal.pgen.1011835)

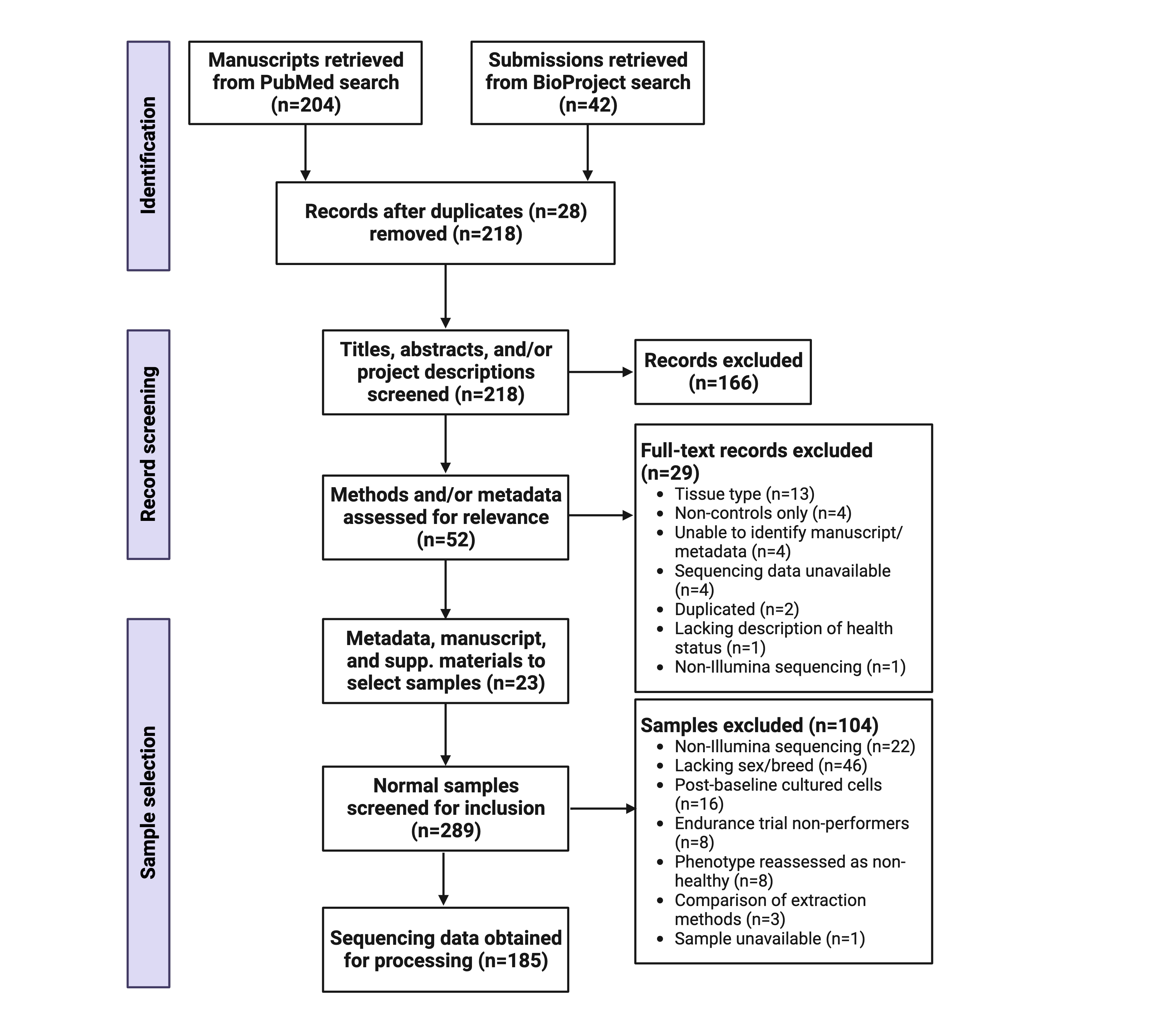

Supplement: S1 Fig — Created with BioRender.com. (TIFF) [file pgen.1011835.s002.tiff]

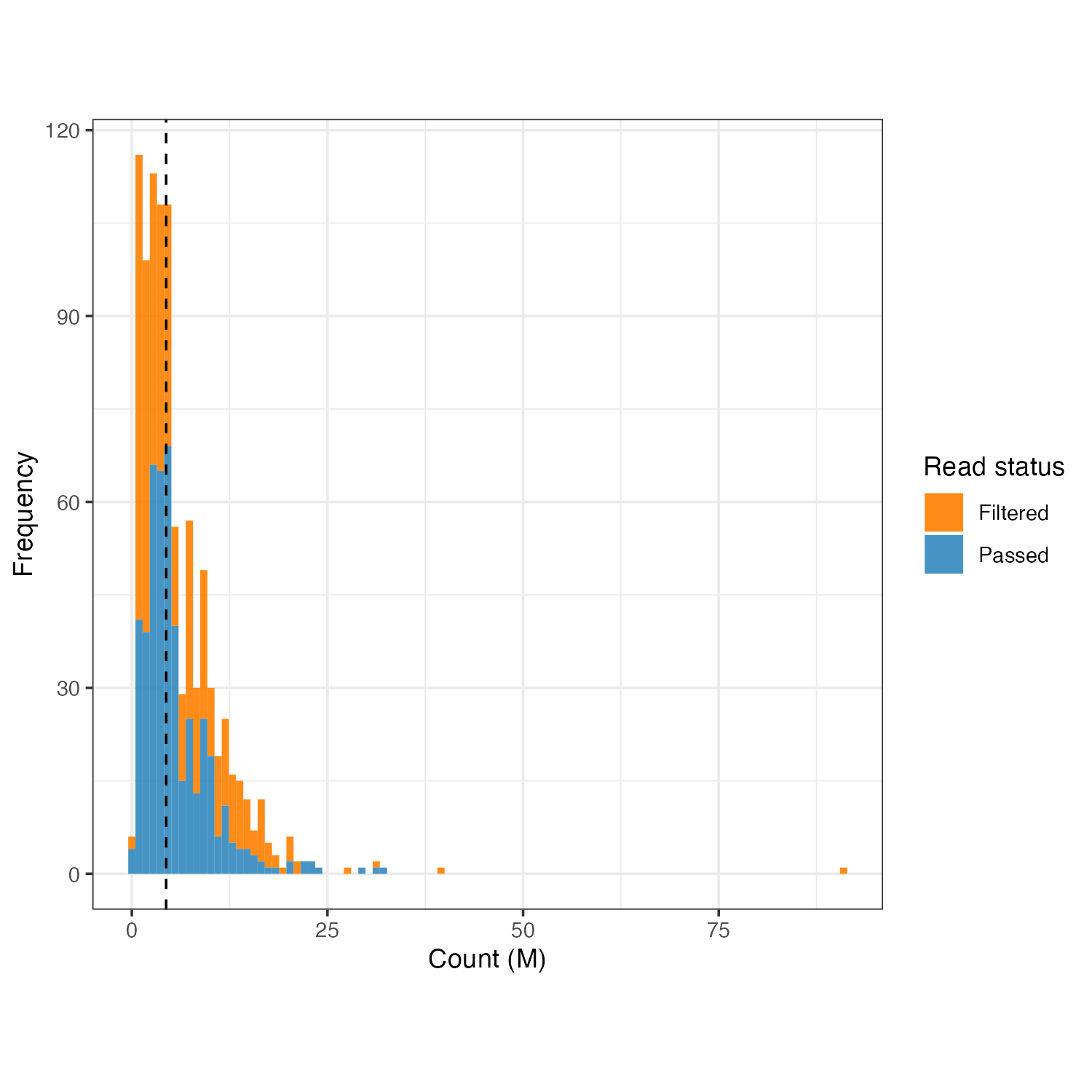

Supplement: S2 Fig — (TIFF) [file pgen.1011835.s003.tiff]

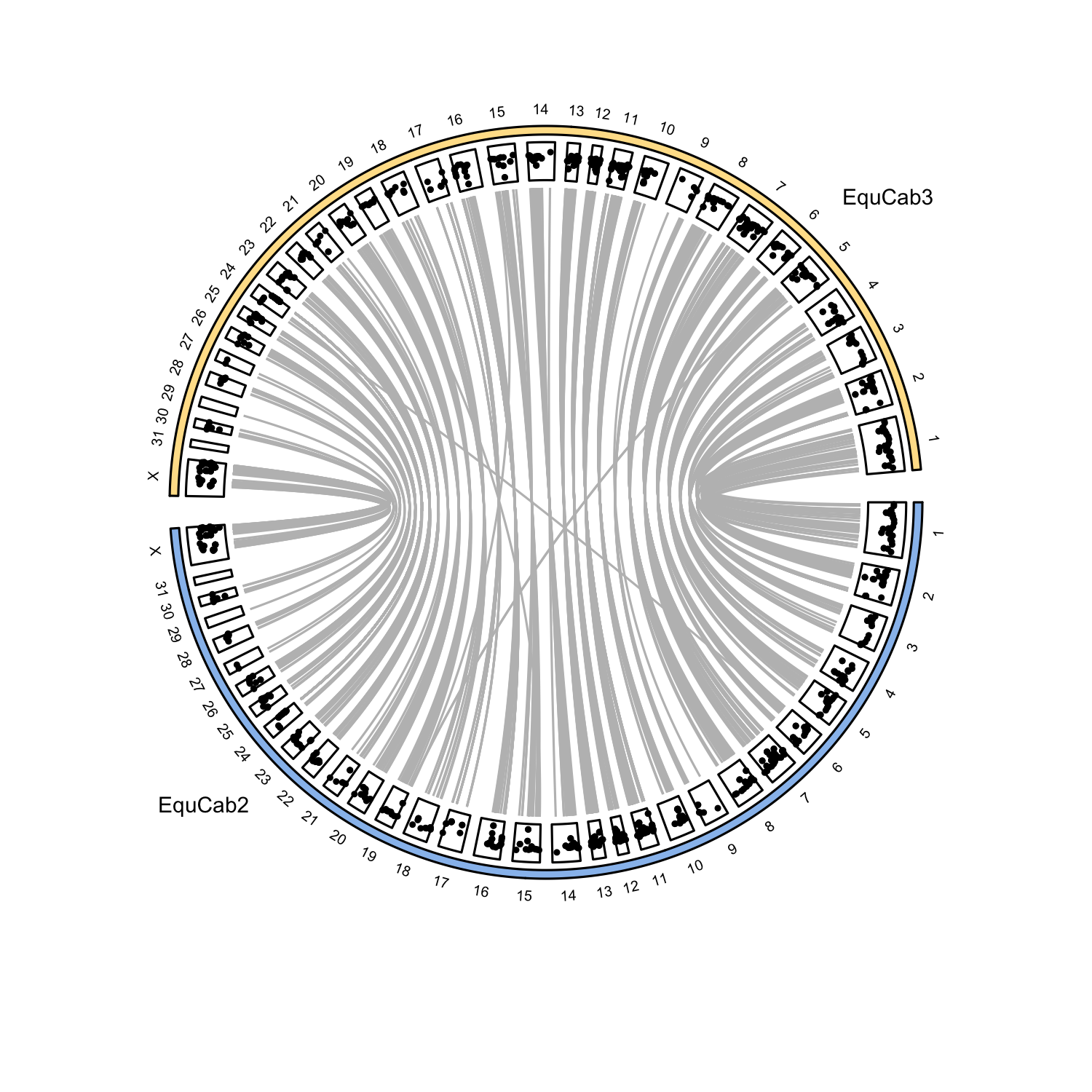

Supplement: S3 Fig — (TIFF) [file pgen.1011835.s004.tiff]
